# Long‐term Results Comparing Cervical Disc Arthroplasty to Anterior Cervical Discectomy and Fusion: A Systematic Review and Meta‐Analysis of Randomized Controlled Trials

**DOI:** 10.1111/os.12585

**Published:** 2019-12-21

**Authors:** Qiao‐li Wang, Zhi‐ming Tu, Pan Hu, Filippos Kontos, Ya‐wei Li, Lei Li, Yu‐liang Dai, Guo‐hua Lv, Bing Wang

**Affiliations:** ^1^ ICU Center The Second Xiangya Hospital of Central South University Changsha China; ^2^ Department of Spine Surgery The Second Xiangya Hospital of Central South University Changsha China; ^3^ Department of Surgery Massachusetts General Hospital, Harvard Medical School Boston Massachusetts USA; ^4^ Department of Orthopaedic Surgery The Third Hospital of Hebei Medical University Shijiazhuang China

**Keywords:** Adjacent segment degeneration, Anterior cervical discectomy and fusion, Cervical disc arthroplasty, Cervical disc disease, Long‐term

## Abstract

**Objective:**

Whether cervical disc arthroplasty (CDA) is superior to anterior cervical discectomy and fusion (ACDF) remains controversial, especially in relation to long‐term results. The present study aimed to evaluate the long‐term safety and efficiency of CDA and ACDF for cervical disc disease.

**Methods:**

We performed this study according to the Cochrane methodology. An extensive search was undertaken in PubMed, Embase, and Cochrane databases up to 1 June 2019 using the following key words: “anterior cervical fusion,” “arthroplasty,” “replacement” and “artificial disc”. RevMan 5.3 (Cochrane, London, UK) was used to analyze data. Safety and efficiency outcome measures included the success rate, functional outcome measures, adverse events (AE), adjacent segment degeneration (ASD), secondary surgery, and patients’ satisfaction and recommendation rates. The OR and MD with 95% confidence interval (CI) were used to evaluate discontinuous and continuous variables, respectively. The statistically significant level was set at *P* < 0.05.

**Results:**

A total of 11 randomized controlled trials with 3505 patients (CDA/ACDF: 1913/1592) were included in this meta‐analysis. Compared with ACDF, CDA achieved significantly higher overall success (2.10, 95% *CI* [1.70, 2.59]), neck disability index (NDI) success (1.73, 95% *CI* [1.37, 2.18]), neurological success (1.65, 95% *CI* [1.24, 2.20]), patients’ satisfaction (2.14, 95% *CI* [1.50, 3.05]), and patients’ recommendation rates (3.23, 95% *CI* [1.79, 5.80]). Functional outcome measures such as visual analog score neck pain (−5.50, 95% *CI* [−8.49, −2.52]) and arm pain (−3.78, 95% *CI* [−7.04, −0.53]), the Short Form‐36 physical component score (SF‐36 PCS) (1.93, 95% *CI* [0.53, 3.32]), and the Short Form‐36 mental component score (SF‐36 MCS) (2.62, 95% *CI* [0.95, 4.29]), revealed superiority in the CDA group. CDA also achieved a significantly lower rate of symptomatic ASD (0.46, 95% *CI* [0.34, 0.63]), total secondary surgery (0.50, 95% *CI* [0.29, 0.87]), secondary surgery at the index level (0.46, 95% *CI* [0.29, 0.74]), and secondary surgery at the adjacent level (0.37, 95% *CI* [0.28, 0.49]). However, no significant difference was found in radiological success (1.35, 95% *CI* [0.88, 2.08]), NDI score (−2.88, 95% *CI* [−5.93, 0.17]), total reported AE (1.14, 95% *CI* [0.92, 1.42]), serious AE (0.89, 95% *CI* [0.71, 1.11]), device/surgery‐related AE (0.90, 95% *CI* [0.68, 1.18]), radiological superior ASD (0.63, 95% *CI* [0.28, 1.43]), inferior ASD (0.45, 95% *CI* [0.19, 1.11]), and work status (1.33, 95% *CI* [0.78, 2.25]). Furthermore, subgroup analysis showed different results between US and non‐US groups.

**Conclusion:**

Our study provided further evidence that compared to ACDF, CDA had a higher long‐term clinical success rate and better functional outcome measurements, and resulted in less symptomatic ASD and fewer secondary surgeries. However, worldwide multicenter RCT with long‐term follow up are still needed for further evaluation in the future.

## Introduction

Anterior cervical discectomy and fusion (ACDF) has been viewed as the gold standard procedure for cervical disc disease (CDD), including radiculopathy and myelopathy. A recent survey revealed that 84.3% of surgeons performed ACDF as the standard technique for CDD^1^. Even though successful clinical outcomes can be achieved with ACDF, postoperative complications such as pseudoarthrosis or non‐union, instrument failure, and adjacent segment degeneration (ASD) have been the greatest concerns^2^, ^3^, ^4^. Cervical fusion could lead to loss of range of motion at the index level and shift load to the adjacent level, then result in accelerating ASD^2^, ^3^, ^5^. Hilibrand *et al*. reported that annually 2.9% of the patients underwent anterior interbody fusion will most likely develop ASD requiring cervical intervention^2^. Thus, spinal surgeons have been attempting to find an alternative procedure to avoid these complications associated with ACDF.

A motion‐preserving procedure, cervical disc arthroplasty (CDA), seems to be a good choice. CDA was initially designed using motion‐preserving techniques to restore cervical physiologic biomechanical properties and alleviate the adjacent‐level loads, and eventually reduces or eliminates the risk of developing ASD^6^. Clinical data showed that preoperative motion could be maintained in the long run following CDA^7^. Promisingly, recent studies have proved that CDA is cost‐effective and is comparable to ACDF in long‐term follow ups^8^, ^9^, ^10^, ^11^. However, some disadvantages of CDA cannot be overlooked, such as heterotopic ossification, implant failure, and bone loss^12^, ^13^, ^14^. In addition, the revision burden of CDA was two times higher than that of ACDF^15^.

In the past 20 years, a series of randomized controlled trials (RCT) have been conducted; however, the reported results are inconsistent and have great variability. Although a few systematic reviews have been performed, researchers have failed to reach an agreement owing to varied criteria^5^, ^16^, ^17^, ^18^, ^19^, ^20^, ^21^, ^22^, ^23^, ^24^. Nevertheless, there is an absence of pooling of long‐term results in a comprehensive meta‐analysis. Therefore, this is the first study aiming at comparing CDA to ACDF with special focus on long‐term safety and efficiency. The conclusions drawn from this study could provide solid evidence for the future application of CDA.

This study was approved by the Ethics Committee of The Second Xiangya Hospital of Centeral South University.

## Methods

### *Literature Search Strategy*

We followed the Cochrane methodology guidelines to perform this meta‐analysis and searched PubMed, Embase, and the Cochrane Central Register of Controlled Trials (CCRCT) databases up to 1 June 2019. The keywords “anterior cervical fusion,” “arthroplasty,” “replacement,” and “artificial disc” combined with “and/or” were used to identify any relevant studies.

### *Inclusion and Exclusion Criteria*

The inclusion criteria were as follows: (i) patients ≥18 years old with symptomatic CDD presenting with radiculopathy and/or myelopathy; (ii) participants were treated with either CDA or ACDF; (iii) comparison was performed between CDA and ACDF; (iv) at least one efficiency and safety outcome measurement was available; and (v) prospective RCT with a follow up ≥5 years.

Articles that met the following characteristics were excluded: (i) reviews, case reports or series, editorials, conference abstracts, and retrospective studies; (ii) duplicated data publications from the same RCT; (iii) partial results with insufficient data; and (iv) non‐English publications.

### *Literature Screening*

Literature screening was performed by two independent investigators (Tu, ZM and Wang, QL). Any disagreement was discussed with another author (Hu, P) to reach consensus. After excluding duplicates, literature selection was carried out according to the inclusion and exclusion criteria based on title and abstract. Then, extensive screening of full‐text articles was performed. All RCT that compared the long‐term efficiency and safety of CDA and ACDF for CDD were included.

### *Quality Assessment of the Included Studies*

Quality assessment was achieved using the criteria recommended by the Cochrane Back Review Group criteria^25^. The types of biases assessed are: four selection bias, four performance bias, two attrition bias, one detection bias, and one reporting bias. The articles scoring at least 6 of these 12 biases were considered as at low risk of bias. The last bia assessed is “Other,” defined as any potential bias not detected using the previous criteria.

### *Data Extraction*

Data extraction was performed as follows: (i) general characteristics such as first author, year of publication, number of clinical trial (NCT), enrolled patients, follow‐up rate, age, sex, surgical levels, type of prosthesis, and follow‐up duration were extracted; and (ii) outcome measures, including clinical success rate (overall success, NDI success, neurological success, and radiological success), functional outcome measurements (NDI score, visual analog score [VAS] neck pain and arm pain, and SF‐36 PCS and MCS), AE (total reported AE, serious AE and device/surgery‐related AE), ASD (symptomatic ASD, radiological superior or inferior ASD), secondary surgery (total secondary surgery, secondary surgery at the index level and at the adjacent level), work status, and patients’ satisfaction and recommendation rates were extracted. This task was performed by two independent investigators (Tu, ZM and Wang, QL), who extracted the data and discussed any disagreement to reach consensus with a third investigator (Hu, P). Data‐extracting software was used to obtain data from figures when original data was not available^26^.

### *Statistical Analysis*

RevMan 5.3 (Cochrane, London, UK) was used to pool extracted data into a combined analysis. The odds ratio (OR) and mean difference (MD) with 95% confidence intervals (CI) were used to evaluate discontinuous and continuous variables, respectively. Heterogeneity was assessed using a χ^2^‐test and an *I* ^2^‐test. A fixed effects model was used when *I* ^2^ < 50%; otherwise, a random effects model was used. Sensitivity analysis was performed by comparing two different effects models. If the statistical difference changed, the leave‐one‐out method^27^ and subgroup analysis was performed to find the origin of heterogeneity. Funnel plots were applied to assess for publication bias. A statistically significant difference was defined as a *P*‐value of less than 0.05.

## Results

### *Literature Review*

**Figure 1:**
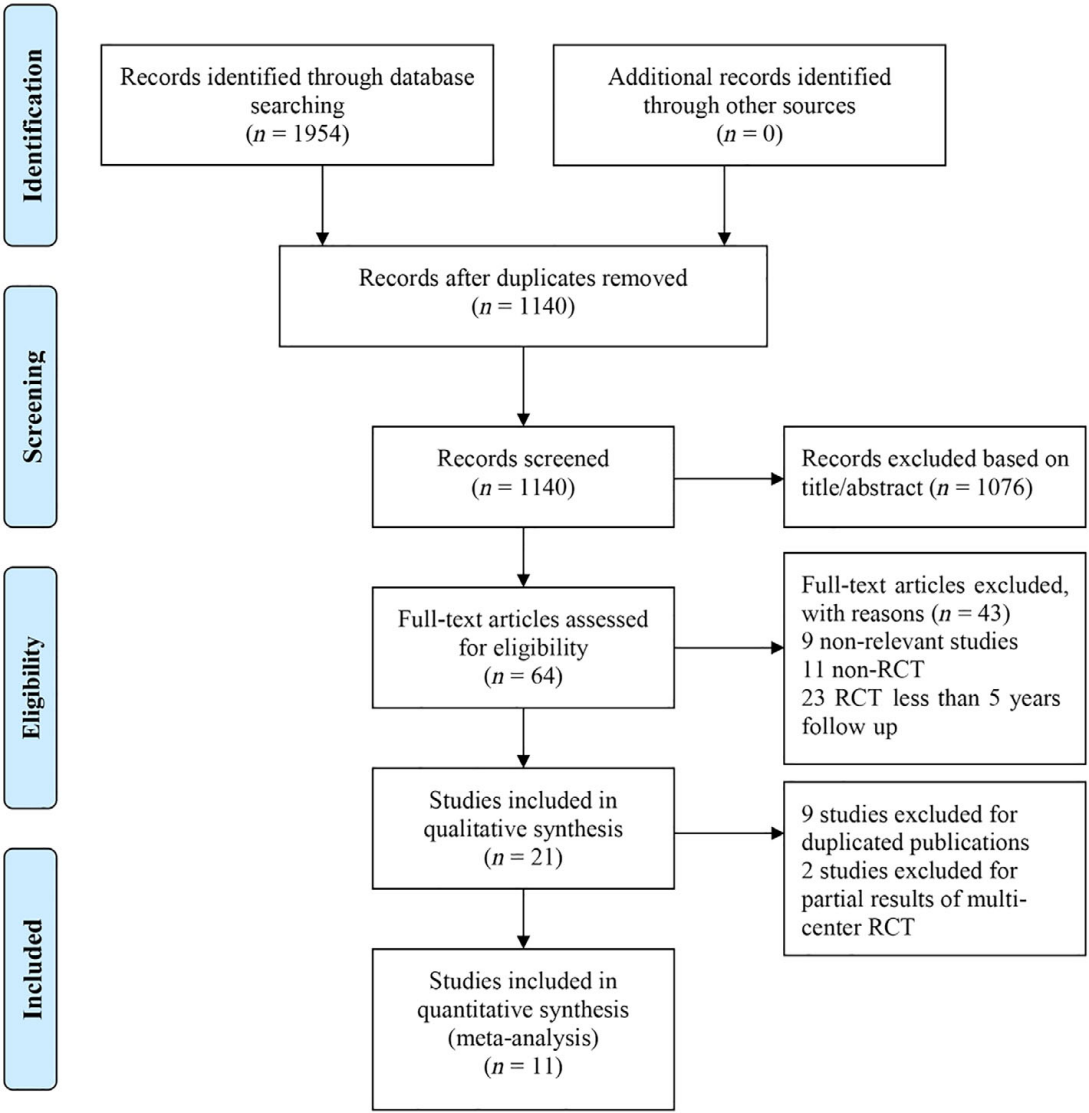
Flow diagram for study selection.

Initial database searching identified 1954 articles (PubMed: 650, Embase: 1020, CCRCT: 284) and detailed literature screening is described in the flow diagram in Figure 1. A total of 814 studies were removed because they were duplicates, 1076 studies were excluded based on their titles and abstracts, and 43 studies were excluded for other reasons. As a result, 21 studies^28^, ^29^, ^30^, ^31^, ^32^, ^33^, ^34^, ^35^, ^36^, ^37^, ^38^, ^39^, ^40^, ^41^, ^42^, ^43^, ^44^, ^45^, ^46^, ^47^, ^48^ were included for further evaluation. Among them, 2 studies^45^, ^47^ were partial results of multicenter RCT and 8 studies^39^, ^40^, ^41^, ^42^, ^43^, ^44^, ^46^, ^48^ included duplicated data for publication. Ultimately, 11 articles^28^, ^29^, ^30^, ^31^, ^32^, ^33^, ^34^, ^35^, ^36^, ^37^, ^38^ involving 3505 patients (CDA/ACDF: 1913/1592) were included in this meta‐analysis. There are 923 male and 990 female patients in the CDA group and 791 male and 801 female patients in the ACDF group. The mean age of each included population varies from 40 to 50 years in both groups. All the patients suffered from radiculopathy and/or myelopathy caused by cervical disc disease with C_3‐4_ to C_6‐7_ involvement. The basic characteristics of the included studies and patients are summarized in Table 1. Among them, 8 studies^28^, ^29^, ^30^, ^32^, ^33^, ^34^, ^36^, ^38^ compared single‐level CDD, 1 study^31^ compared two‐level CDD, and 2 studies^35^, ^37^ compared both single‐level and two‐level CDD independently.

**Table 1:** Characteristics of included studies

Study	Region	Number of clinical trial	Design	Enrolled patients (CDA/ACDF)	Follow‐up rate (CDA/ACDF)	Age (CDA/ACDF)	Sex (female)	BMI (CDA/ACDF)	Level	Prosthesis	Follow up
Burkus 2014^28^	USA	NCT00642876	RCT, 31‐sites	276/265	76.8%/69.1%	43.3/43.9	148/142	/	1	Prestige ST	7 years
Coric 2018^29^	USA	NCT00374413	RCT, 21‐sites	136/133	68.4%/62.4%	43.7(7.76)/43.9(7.39)	74/74	27.5(5.0)/28.7(5.7)	1	Kineflex|C	5 years
Donk 2017^30^	Netherlands	ISRCTN41681847	RCT, single‐site	50/47	98.0%/97.9%	44.1(6.4)/43.1(7.5)	26/22	/	1	Bryan	9 years
Gornet 2019^31^	USA	NCT00637156	RCT, 30‐sites	209/188	86.0%/84.9%	47.1(8.3)/47.3(7.7)	117/98	28.2(5.6)/28.6(4.9)	2	Prestige LP	10 years
Hou 2016^32^	China	Unknown	RCT, single‐site	56/51	91.1%/94.1%	46.3(7.8)/48.5(8.3)	30/28	21(3.2)/22(2.5)	1	Mobi‐C	5 years
Janssen 2015^33^	U.S.	NCT00291018	RCT, 13‐sites	103/106	92%/92%	42.1(8.42)/43.5(7.15)	55/54	26.44(5.32)/27.34(5.54)	1	ProDisc‐C	7 years
Lavelle 2018^34^	USA	NCT00437190	RCT, 38‐sites	242/221	100%/100%	44.4/44.7	132/108	26.6(4.8)/27.6(5.0)	1	BRYAN	10 years
MacDowall 2019^35^	Sweden	ISRCTN44347115	RCT, 3‐sites	83/70	89.2%/87.1	46.9 (6.8)/47.0 (6.9)	42/33	26/26	1–2	Discover	5 years
Phillips 2015^36^	USA	Unknown	RCT, 24‐sites	218/185	74.8%/70.3%	49.3(5.0)/43.7(8.3)	105/89	28.2(4.6)/27.4(4.8)	1	PCM	5 years
Radcliff 2017a^37^ *	USA	NCT00389597	RCT, 24‐sites	164/81	80.1%/74.3%	43.3(9.2)/44.0(8.2)	78/36	27.3 (4.4)/27.4 (4.2)	1	Mobi‐C	7 years
Radcliff 2017b^37^	USA	NCT00389597	RCT, 24‐sites	225/105	84.4%/75%	44.3(8.1)/46.2(8.0)	113/45	27.6(4.5)/28.1(4.2)	2	Mobi‐C	7 years
Vaccaro 2018^38^	USA	Unknown	RCT, 18‐sites	151/140	86.1%/84.2%	43.4(7.50)/44.4(7.86)	70/72	28.9(5.53)/29.0(5.47)	1	SECURE‐C	7 years

* Radcliff (2017a) and Radcliff (2017b) are from the same study.

ACDF, anterior cervical discectomy and fusion; BMI, body mass index; CDA, cervical disc arthroplasty.

### *Quality Assessment of the Included Studies*

**Figure 2:**
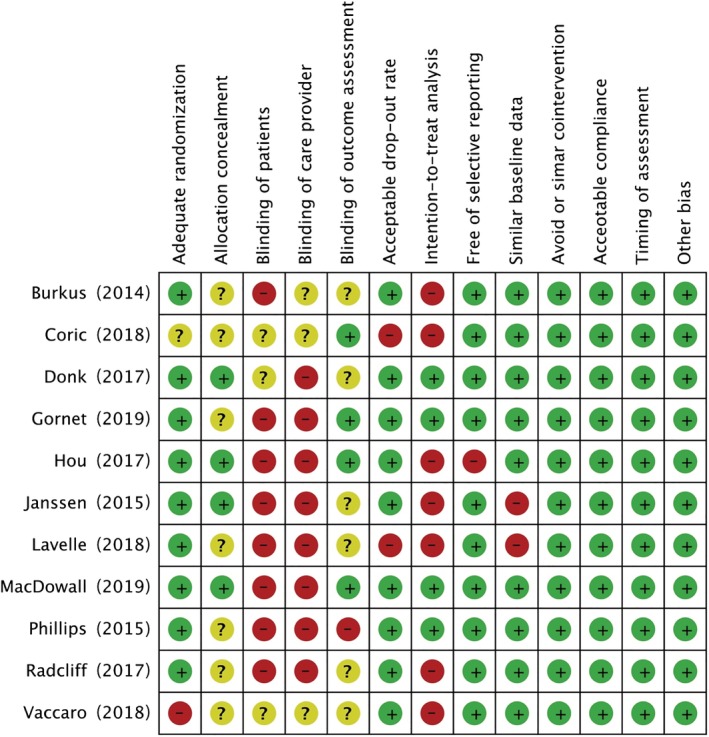
Risk bias of included studies.

Methodological quality assessment of the 11 eligible studies is shown in Fig. 2. Nine studies^28^, ^30^, ^31^, ^32^, ^33^, ^34^, ^35^, ^36^, ^37^ were adequately randomized, but 1 study^29^ did not provide detailed information of randomization, and 1 study^38^ failed to achieve adequate randomization. Only 4 studies^30^, ^32^, ^33^, ^35^ provided a clear statement regarding avoiding allocation concealment. In addition, all included RCT^28^, ^29^, ^30^, ^31^, ^32^, ^33^, ^34^, ^35^, ^36^, ^37^, ^38^ failed to achieve blinding to patients and care providers due to the specialty of this kind of trial. The patients were informed immediately after surgery about the type of surgical procedure they had been underwent, and care providers were aware of which kind of surgery was to be performed during surgery^28^, ^30^, ^31^, ^32^, ^33^, ^34^, ^35^, ^36^, ^37^. Almost all the studies described the dropout rate and 2 studies^28^, ^29^ with a follow‐up rate below 70% were considered as having high risk of bias. All included studies were scored above seven and were rated as having low risk of bias.

### *Heterogeneity Analysis*

Of all the parameters identified for meta‐analysis, 6 studies compared overall success^28^, ^29^, ^31^, ^34^, ^37^, ^38^ and NDI success^28^, ^31^, ^34^, ^36^, ^37^, ^38^, 7 studies compared neurological success^28^, ^31^, ^33^, ^34^, ^36^, ^37^, ^38^, 3 studies compared radiological success^31^, ^36^, ^37^, 7 studies compared NDI score^28^, ^32^, ^33^, ^35^, ^36^, ^37^, ^38^, 5 studies compared neck pain score^33^, ^35^, ^36^, ^37^, ^38^, 4 studies compared arm pain score 33, 35, 36, 37, 5 studies compared SF‐36 PCS^28^, ^30^, ^33^, ^36^, ^38^, 4 studies compared SF‐36 MCS^30^, ^33^, ^36^, ^38^, 8 studies compared any AE^28^, ^29^, ^30^, ^31^, ^33^, ^36^, ^37^, ^38^, 4 studies compared serious AE^31^, ^36^, ^37^, ^38^, 6 studies compared device/surgery‐related AE^29^, ^31^, ^33^, ^36^, ^37^, ^38^ and symptomatic ASD^30^, ^33^, ^34^, ^35^, ^37^, ^38^, 2 studies compared radiological superior and inferior ASD^37^, ^38^, 8 studies compared total secondary surgeries^28^, ^29^, ^30^, ^32^, ^33^, ^35^, ^36^, ^37^ and secondary surgeries at the index level^28^, ^30^, ^31^, ^33^, ^35^, ^36^, ^37^, ^38^, 9 studies compared secondary surgeries at the adjacent level^28^, ^30^, ^31^, ^33^, ^34^, ^35^, ^36^, ^37^, ^38^, 2 studies compared work status^28^, ^34^, 4 studies compared patients’ satisfaction rate^31^, ^36^, ^37^, ^38^, and 2 studies compared patients’ recommendation rate^36^, ^37^.

The heterogeneity test showed that *I* ^2^ < 50% for overall success, NDI success, neurological success, radiological success, VAS neck pain and arm pain, SF‐36 PCS and MCS, total reported AE, serious AE, device/surgery‐related AE, symptomatic ASD, secondary surgery at the adjacent level, and patients’ satisfaction and recommendation rates. This indicates that there is low heterogeneity among these parameters and a fix effects model could be applied for combined statistics. In contrast, the heterogeneity test showed *I* ^2^ > 50% for NDI score, radiological superior and inferior ASD, total secondary surgery, secondary surgery at the index level, and work status, which indicates significant or large heterogeneity. Therefore, a random effects model could be applied for combined statistics. The results of the heterogeneity test are summarized in Table 2.

**Table 2:** The heterogeneity test and meta‐analysis of outcome measurements

Outcome measurements	Included studies	Participants	*I* ^2^	Statistic effect model	Effect estimate	*P‐*value
Overall success	6	1734	0%	*OR* (M‐H, Fixed, 95% *CI*)	2.10 [1.70, 2.59]	<0.00001
NDI success	6	1972	20%	*OR* (M‐H, Fixed, 95% *CI*)	1.73 [1.37, 2.18]	<0.00001
Neurological success	7	1982	16%	*OR* (M‐H, Fixed, 95% CI)	1.65 [1.24, 2.20]	0.0006
Radiological success	3	1002	0%	*OR* (M‐H, Fixed, 95% *CI*)	1.35 [0.88, 2.08]	0.17
NDI score	7	1885	68%	*MD* (IV, Random, 95% *CI*)	−2.88 [−5.93, 0.17]	0.06
VAS neck pain	5	1366	33%	*MD* (IV, Fixed, 95% *CI*)	−5.50 [−8.49, −2.52]	0.0003
VAS arm pain	4	1134	0%	*MD* (IV, Fixed, 95% *CI*)	−3.78 [−7.04, −0.53]	0.02
SF‐36 PCS	4	1149	0%	*MD* (IV, Fixed, 95% *CI*)	1.93 [0.53, 3.32]	0.007
SF‐36 MCS	3	761	0%	*MD* (IV, Fixed, 95% CI)	2.62 [0.95, 4.29]	0.002
Total reported AE	8	2872	46%	*OR* (M‐H, Fixed, 95% *CI*)	1.14 [0.92, 1.42]	0.22
Serious AE	4	1756	13%	*OR* (M‐H, Fixed, 95% *CI*)	0.89 [0.71, 1.11]	0.29
Device/surgery‐related AE	6	2317	2%	*OR* (M‐H, Fixed, 95% *CI*)	0.90 [0.68, 1.18]	0.43
Symptomatic ASD	6	1628	29%	*OR* (M‐H, Fixed, 95% *CI*)	0.46 [0.34, 0.63]	<0.00001
Radiological superior ASD	2	659	83%	*OR* (M‐H, Random, 95% *CI*)	0.63 [0.28, 1.43]	0.27
Radiological inferior ASD	2	474	78%	*OR* (M‐H, Random, 95% *CI*)	0.45 [0.19, 1.11]	0.08
Total secondary surgery	8	2058	64%	*OR* (M‐H, Random, 95% *CI*)	0.50 [0.29, 0.87]	0.01
Secondary surgery at the index level	8	2712	55%	*OR* (M‐H, Random, 95% *CI*)	0.46 [0.29, 0.74]	0.001
Secondary surgery at the adjacent level	9	2937	18%	*OR* (M‐H, Fixed, 95% *CI*)	0.37 [0.28, 0.49]	<0.00001
Work status	2	622	53%	*OR* (M‐H, Random, 95% *CI*)	1.33 [0.78, 2.25]	0.29
Patients’ satisfaction rate	4	1224	0%	*OR* (M‐H, Fixed, 95% *CI*)	2.14 [1.50, 3.05]	<0.0001
Patients’ recommendation rate	2	727	0%	*OR* (M‐H, Fixed, 95% *CI*)	3.23 [1.79, 5.80]	<0.0001

AE, adverse event; ASD, adjacent segment degeneration; *CI*, confidence interval; MD, mean difference; NDI, neck disability index; *OR*, odds ratio; VAS, visual analog score.

### *Results of the Meta‐Analysis*

**Figure 3:**
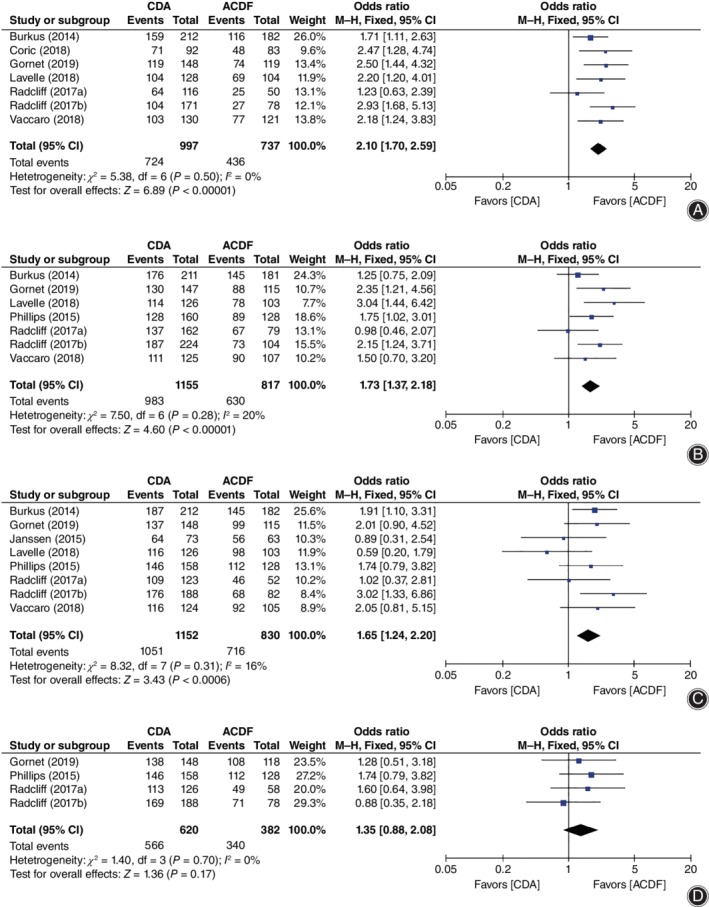
Forest plot comparing clinical success rate between cervical disc arthroplasty (CDA) and anterior cervical discectomy and fusion (ACDF). (A) Overall success. (B) Neck disability index (NDI) success. (C) Neurological success. (D) Radiological success. *CI*, confidence interval.

**Figure 4:**
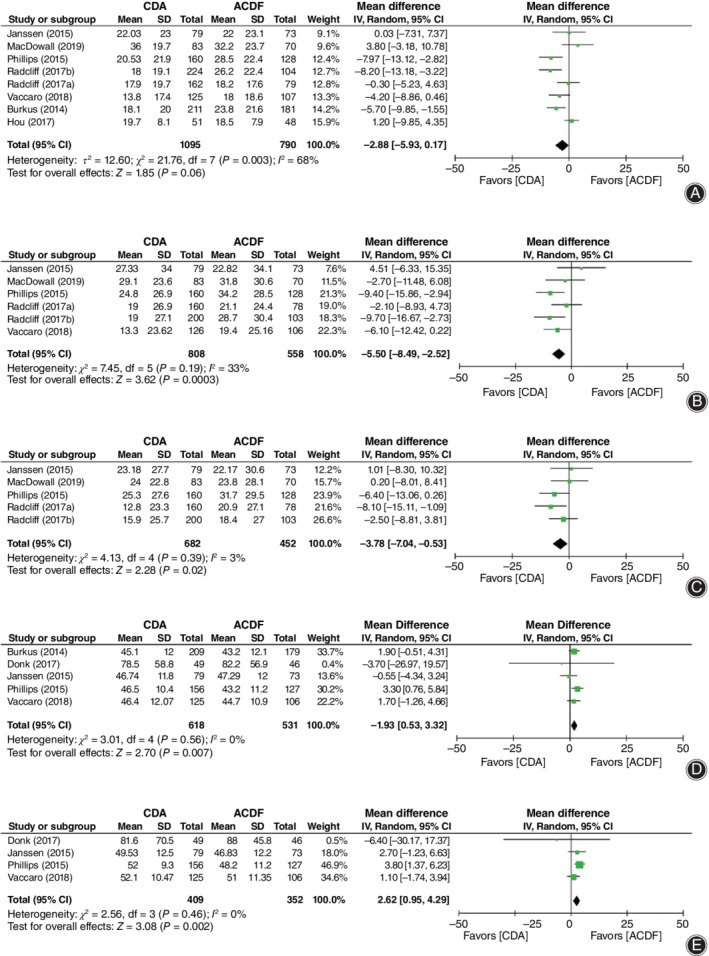
Forest plot comparing functional outcome measurements between cervical disc arthroplasty (CDA) and anterior cervical discectomy and fusion (ACDF). (A) Neck disability index (NDI) score. (B) Visual analog score (VAS) neck pain. (C) VAS arm pain. (D) Short Form‐36 physical component score (SF‐36 PCS). (E) Short Form‐36 mental component score (SF‐36 MCS). *CI*, confidence interval.

**Figure 5:**
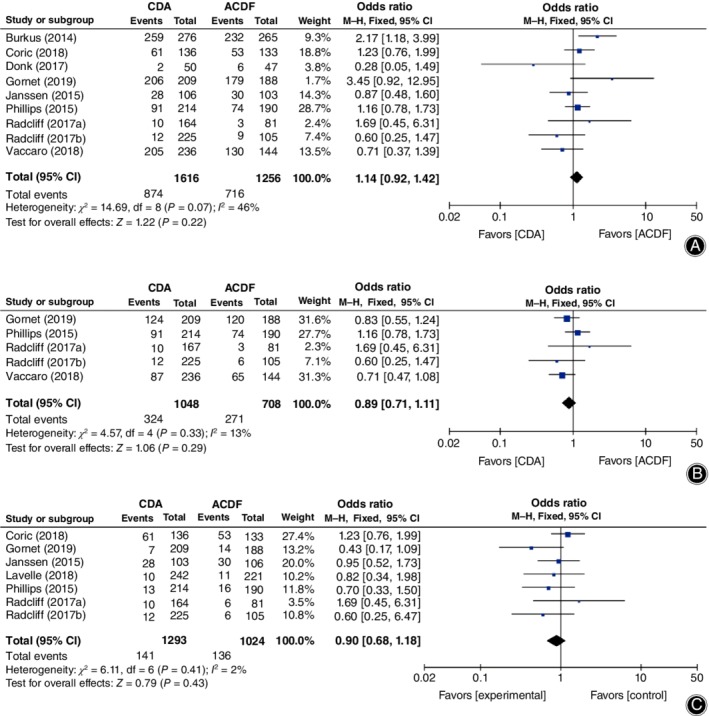
Forest plot showing a comparison of the frequency of adverse events (AE) between cervical disc arthroplasty (CDA) and anterior cervical discectomy and fusion (ACDF). (A) Total reported AE. (B) Serious AE. (C) Device/surgery‐related AE. *CI*, confidence interval.

**Figure 6:**
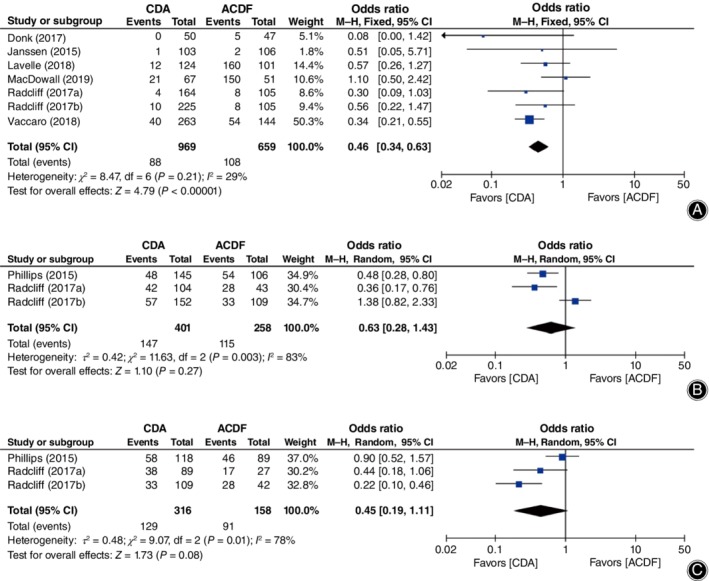
Forest plot comparing the incidence of adjacent segment degeneration (ASD) between cervical disc arthroplasty (CDA) and anterior cervical discectomy and fusion (ACDF). (A) Symptomatic ASD. (B) Radiological superior ASD. (C) Radiological inferior ASD. *CI*, confidence interval.

**Figure 7:**
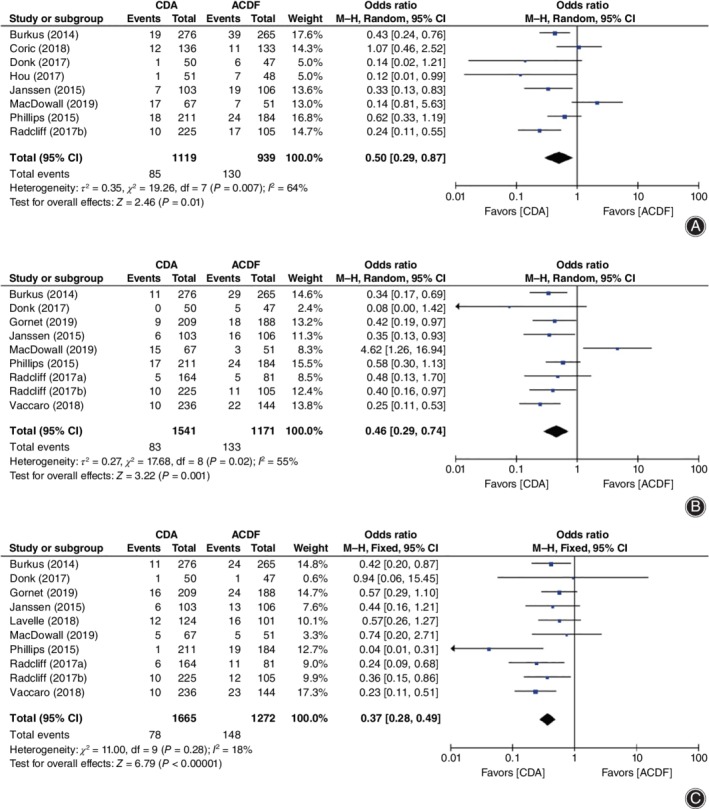
Forest plot showing a comparison of secondary surgery rate between cervical disc arthroplasty (CDA) and anterior cervical discectomy and fusion (ACDF). (A) Total secondary surgery. (B) Secondary surgery at the index level. (C) Secondary surgery at the adjacent level. *CI*, confidence interval.

**Figure 8:**
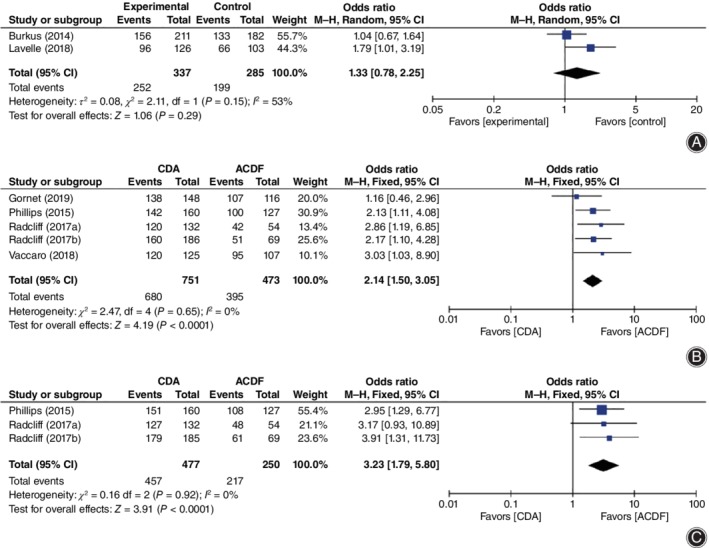
Forest plot comparing work status (A), patients’ satisfaction (B), and patients’ recommendation (C) between cervical disc arthroplasty (CDA) and anterior cervical discectomy and fusion (ACDF). *CI*, confidence interval.

We pooled all extracted data comparing CDA with ACDF for CDD in this meta‐analysis. The combined results are shown in Table 2 and Figs 3, 4, 5, 6, 7, 8.

For clinical success rate, CDA showed significant superiority in overall success (*OR* = 2.10, 95% *CI* [1.70, 2.59], *P* < 0.00001, Fig. 3A), NDI success (*OR* = 1.73, 95% *CI* [1.37, 2.18], *P* < 0.00001; Fig. 3B), and neurological success (*OR* = 1.65, 95% *CI* [1.24, 2.20], *P* = 0.0006; Fig. 3C), while no superiority was found in radiological success (*OR* = 1.35, 95% *CI* [0.88, 2.08], *P* = 0.17; Fig. 3D).

Functional outcome measurements showed superiority in CDA except for NDI score. The NDI score (*WMD* = −2.88, 95% *CI* [−5.93, 0.17]), *P* = 0.06; Fig. 4A) was found to be lower in CDA without statistical difference. However, the combined results that favored CDA were identified in neck pain score (*WMD* = −5.50, 95% *CI* [−8.49, −2.52], *P* = 0.0003; Fig. 4B), arm pain score (*WMD* = −3.78, 95% *CI* [−7.04, −0.53], *P* = 0.02; Fig. 4C), SF‐36 PCS (*WMD* = 1.93, 95% *CI* [0.53, 3.32], *P* = 0.0007; Fig. 4D), and SF‐36 MCS (*WMD* = 2.62, 95% *CI* [0.95, 4.29], *P* = 0.002; Fig. 4E).

No superiority was showed in AE. Total reported AE (*OR* = 1.14, 95% *CI* [0.92, 1.42], *P* = 0.22, Fig. 5A), serious AE (*OR* = 0.89, 95% *CI* [0.71, 1.11], *P* = 0.29, Fig. 5B), and device/surgery‐related AE (*OR* = 0.90, 95% *CI* [0.68, 1.18], *P* = 0.43; Fig. 5C) were similar between CDA and ACDF.

As for ASD, the incidence of symptomatic ASD (*OR* = 0.46, 95% *CI* [0.34, 0.63]), *P* < 0.00001; Fig. 6A) was significantly lower in CDA; however, radiologically superior ASD (*OR* = 0.63, 95% *CI* [0.28, 1.43], *P* = 0.27; Fig. 6B) and inferior ASD (*OR* = 0.45, 95% *CI* [0.19, 1.11], *P* = 0.08; Fig. 6C) were not significantly different between groups.

Strikingly, when compared to ACDF, our results revealed that CDA had significant superiority in total secondary surgery (*OR* = 0.50, 95% *CI* [0.29, 0.87], *P* = 0.01, Fig. 7A), secondary surgery at the index level (*OR* = 0.46, 95% *CI* [0.29, 0.74], *P* = 0.001, Fig. 7B), and secondary surgery at the adjacent level (*OR* = 0.37, 95% *CI* [0.28, 0.49], *P* < 0.00001; Fig. 7C).

Finally, work status (*OR* = 1.33, 95% *CI* [0.78, 2.25], *P* = 0.29, Fig. 8A) was similar at the last follow up between CDA and ACDF. CDA achieved a higher rate of patient satisfaction (*OR* = 2.14, 95% *CI* [1.50, 3.05], *P* = 0.0002; Fig. 8B) and patients’ recommendation (*OR* = 3.23, 95% *CI* [1.79, 5.80], *P* < 0.00001; Fig. 8C).

### *Sensitivity Analysis*

Combined OR or MD with 95% CI using fixed and random effects for all outcome measures are showed in Table 3. The consistency of the combined results was identified in overall success, NDI success, neurological success, radiological success, VAS neck pain and arm pain, SF‐36 PCS and MCS, total reported AE, serious AE, device/surgery‐related AE, symptomatic ASD, total secondary surgery, secondary surgery at the index level and at the adjacent level, and patients’ satisfaction and recommendation rates. This means that these results are stable and reliable. However, the situation was quite different for NDI score, and radiological superior and inferior ASD, indicating that the combined results were unreliable. Therefore, further analysis was performed.

**Table 3:** Comparison of the combined results from fixed and random effects model

	Fixed effects model		Random effects model	
Outcome measures	Effect estimated	*P*‐value	Effect estimated	*P‐*value
Overall success	2.10 [1.70, 2.59]	<0.00001	2.10 [1.70, 2.59]	<0.00001
NDI success	1.73 [1.37, 2.18]	<0.00001	1.73 [1.33, 2.26]	<0.00001
Neurological success	1.65 [1.24, 2.20]	0.0006	1.64 [1.19, 2.27]	0.003
Radiological success	1.35 [0.88, 2.08]	0.17	1.36 [0.87, 2.10]	0.17
NDI score	−2.67 [−4.33, −1.01]	0.002	−2.88 [−5.93, 0.17]	0.06
VAS neck pain	−5.50 [−8.49, −2.52]	0.0003	−5.21 [−8.91, −1.51]	0.006
VAS arm pain	−3.78 [−7.04, −0.53]	0.02	−3.77 [−7.08, −0.46]	0.03
SF‐36 PCS	1.93 [0.53, 3.32]	0.007	1.93 [0.53, 3.32]	0.007
SF‐36 MCS	2.62 [0.95, 4.29]	0.002	2.62 [0.95, 4.29]	0.002
Total reported AE	1.14 [0.92, 1.42]	0.22	1.12 [0.80, 1.55]	0.51
Serious AE	0.89 [0.71, 1.11]	0.29	0.88 [0.69, 1.13]	0.32
Device/surgery‐related AE	0.90 [0.68, 1.18]	0.43	0.89 [0.67, 1.18]	0.42
Symptomatic ASD	0.46 [0.34, 0.63]	<0.00001	0.49 [0.32, 0.76]	0.001
Radiological superior ASD	0.69 [0.50, 0.95]	0.02	0.63 [0.28, 1.43]	0.27
Radiological inferior ASD	0.53 [0.36, 0.78]	0.001	0.45 [0.19, 1.11]	0.08
Total secondary surgery	0.52 [0.39, 0.69]	<0.00001	0.50 [0.29, 0.87]	0.01
Secondary surgery at the index level	0.46 [0.34, 0.61]	<0.00001	0.46 [0.29, 0.74]	0.001
Secondary surgery in the adjacent level	0.37 [0.28, 0.49]	<0.00001	0.39 [0.28, 0.55]	<0.00001
Work status	1.28 [0.90, 1.82]	0.17	1.33 [0.78, 2.25]	0.29
Patients’ satisfaction rate	2.14 [1.50, 3.05]	<0.0001	2.14 [1.50, 3.06]	<0.0001
Patients’ recommendation rate	3.23 [1.79, 5.80]	<0.0001	3.25 [1.81, 5.82]	<0.0001

AE, adverse event; ASD, adjacent segment degeneration; CI, confidence interval; NDI, neck disability index; VAS, visual analog score.

Then, we performed sensitivity analysis based on the leave‐one‐out method^27^. For NDI score, we found that the combined result changed significantly when removing the study from Hou *et al*.^32^ or MacDowall *et al*.^35^, with the *P*‐value reduced from 0.06 to 0.02. Thus, we performed a subgroup analysis (Table 4) and found that the heterogeneity was 40% and 0% in the US and non‐US subgroups, respectively, indicating that the heterogeneity originated from the studies from different regions. In addition, for radiological superior ASD, after we excluded the data from Radicliff *et al*. (2017)^37^, *I* ^2^ decreased from 83% to 0%, and the statistical significance changed. For radiological inferior ASD, after we excluded the study from Phillips *et al*.^36^, *I* ^2^ decreased from 78% to 28%, and the statistical significance also changed. This indicates that they were the source of heterogeneity for radiological superior and inferior ASD, respectively.

**Table 4:** The combined results of subgroup analysis based on regions

Outcome measurements		Included studies	Participants	*I* ^2^	Statistic effect model	Effect estimate	*P*‐value
NDI score	US	6	1633	40%	*MD* (IV, Random, 95% *CI*)	−4.71 [−7.38, −2.04]	0.0005
	Non‐US	2	252	0%	*MD* (IV, Fixed, 95% *CI*)	1.64 [−1.23, 4.51]	0.26
Symptomatic ASD	US	5	1413	0%	*OR* (M‐H, Fixed, 95% *CI*)	0.40 [0.28, 0.58]	<0.00001
	Non‐US	2	215	68%	*OR* (M‐H, Random, 95% *CI*)	0.42 [0.03, 5.57]	0.51
Total secondary surgery	US	5	1744	47%	*OR* (M‐H, Fixed, 95% *CI*)	0.48 [0.35, 0.66]	<0.00001
	Non‐US	3	314	79%	*OR* (M‐H, Random, 95% *CI*)	0.39 [0.04, 3.49]	=0.40
Secondary surgery at the index level	US	7	2497	0%	*OR* (M‐H, Fixed, 95% *CI*)	0.39 [0.29, 0.53]	<0.00001
	Non‐US	2	215	85%	*OR* (M‐H, Random, 95% *CI*)	0.73 [0.01, 46.24]	=0.88
Secondary surgery at the adjacent level	US	8	2722	27%	*OR* (M‐H, Fixed, 95% CI)	0.35 [0.26, 0.47]	<0.00001
	Non‐US	2	215	0%	*OR* (M‐H, Fixed, 95% *CI*)	0.77 [0.24, 2.51]	=0.67

ASD, adjacent segment degeneration; CI, confidence interval; MD, mean difference; NDI, neck disability index; OR, odds ratio.

### *Subgroup Analysis*

First, we performed subgroup analysis based on different regions. The included studies were classified into US and non‐US subgroups. The combined results of NDI score, symptomatic ASD, total secondary surgery, and secondary surgery at the index level and at the adjacent level are shown in Table 4. Surprisingly, the combined results showed that CDA was superior to ACDF, with significant difference in all these outcome measures in the US subgroup. However, in the non‐US subgroup, all these combined results were similar without statistical difference.

Second, we performed subgroup analysis based on the number of surgical levels. The combined results of overall success, neurological success, NDI success, radiological success, total reported AE, serious AE, device/surgery‐related AE, secondary surgery at the index level and at the adjacent level, and patients’ satisfaction rate are showed in Table 5. The combined results showed significantly less device/surgery‐related AE of CDA in the two‐level CDD group, with no statistical difference in single‐level CDD. In contrast, patients’ satisfaction favored CDA in single‐level CDD (*P* = 0.0002), while in two‐level CDD (*P* = 0.05), further studies are needed to identify the superiority. The residual outcome measures are similar for single‐level and two‐level CDD.

**Table 5:** The combined results of subgroup analysis based on surgical level

Outcome measurements		Included studies	Participants	*I* ^2^	Statistic effect model	Effect estimate	*P*‐value
Overall success	Single‐level	5	1218	0%	*OR* (M‐H, Fixed, 95% *CI*)	1.89 [1.47, 2.42]	<0.00001
	Two‐level	2	516	0%	*OR* (M‐H, Fixed, 95% *CI*)	2.70 [1.83, 4.00]	<0.00001
NDI success	Single‐level	5	1382	27%	*OR* (M‐H, Fixed, 95% *CI*)	1.55 [1.17, 2.05]	0.002
	Two‐level	2	590	0%	*OR* (M‐H, Fixed, 95% *CI*)	2.23 [1.46, 3.40]	0.0002
Neurological success	Single‐level	6	1449	9%	*OR* (M‐H, Fixed, 95% *CI*)	1.46 [1.04, 2.03]	0.03
	Two‐level	2	533	0%	*OR* (M‐H, Fixed, 95% *CI*)	2.44 [1.37, 4.34]	0.003
Radiological success	Single‐level	2	470	0%	*OR* (M‐H, Fixed, 95% *CI*)	1.68 [0.92, 3.05]	0.09
	Two‐level	2	532	0%	*OR* (M‐H, Fixed, 95% *CI*)	1.06 [0.56, 2.00]	0.87
Total reported AE	Single‐level	7	2145	40%	*OR* (M‐H, Fixed, 95% *CI*)	1.14 [0.91, 1.43]	0.24
	Two‐level	2	727	79%	*OR* (M‐H, Random, 95% *CI*)	1.34 [0.24, 7.49]	0.74
Serious AE	Single‐level	3	1029	43%	*OR* (M‐H, Fixed, 95% *CI*)	0.95 [0.72, 1.26]	0.72
	Two‐level	2	727	0%	*OR* (M‐H, Fixed, 95% *CI*)	0.79 [0.54, 1.14]	0.20
Device/surgery‐related AE	Single‐level	5	1590	0%	*OR* (M‐H, Fixed, 95% CI)	1.02 [0.75, 1.38]	0.91
	Two‐level	2	727	0%	*OR* (M‐H, Fixed, 95% CI)	0.51 [0.27, 0.96]	0.04
Secondary surgery at the index level	Single‐level	6	1867	0%	*OR* (M‐H, Fixed, 95% *CI*)	0.37 [0.26, 0.52]	<0.00001
	Two‐level	2	727	0%	*OR* (M‐H, Fixed, 95% *CI*)	0.41 [0.22, 0.75]	0.004
Secondary surgery at the adjacent level	Single‐level	7	2092	29%	*OR* (M‐H, Fixed, 95% *CI*)	0.31 [0.22, 0.45]	<0.00001
	Two‐level	2	727	0%	*OR* (M‐H, Fixed, 95% *CI*)	0.48 [0.28, 0.82]	0.007
Patients’ satisfaction rate	Single‐level	3	705	0%	*OR* (M‐H, Fixed, 95% *CI*)	2.48 [1.55, 3.96]	0.0002
	Two‐level	2	519	12%	*OR* (M‐H, Fixed, 95% *CI*)	1.73 [1.00, 3.00]	0.05

AE, adverse event; *CI*, confidence interval; NDI, neck disability index; *OR*, odds ratio

### *Assessment of Publication Bias*

**Figure 9:**
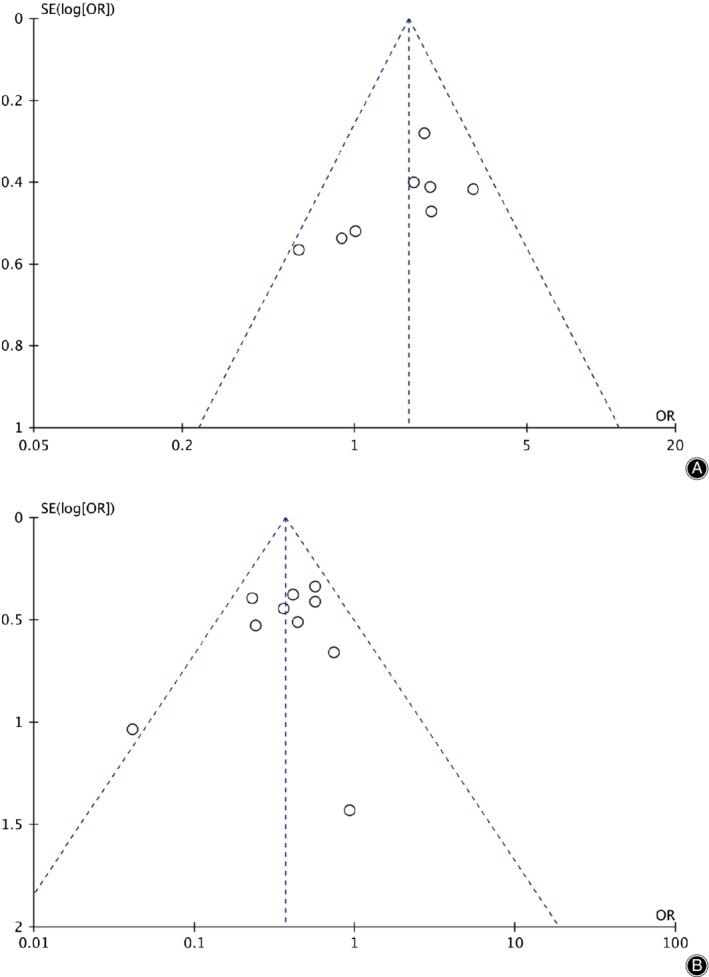
The funnel plot of neurological success (A) and secondary surgery at the adjacent level (B). CI, confidence interval.

The funnel plot was applied to detect publication bias. As for neurological success (Fig. 9A), the funnel plots appeared symmetric and all studies were included inside, indicating that no publication bias existed. However, for secondary surgery at the adjacent level (Fig. 9B), the funnel plots appeared symmetric and 1 study was not included inside, indicating that publication bias existed.

## Discussion

Up to now, CDA application in spinal practice has remained controversial. Whether CDA is superior to ACDF has not been established in the long run ASD is always associated with the length of follow up. Therefore, it is crucial to evaluate the safety and efficiency of CDA in the long run. To our knowledge, there have been several meta‐analyses comparing CDA with ACDF. Most of them have included partial long‐term results, but they were mixed up with short‐term and mid‐term results^5^, ^16^, ^17^, ^18^, ^19^, ^20^, ^21^, ^22^, ^23^. Therefore, given the availability of newly published long‐term results^28^, ^29^, ^30^, ^31^, ^32^, ^33^, ^34^, ^35^, ^36^, ^37^, ^38^, we performed this study. This is the first time comparing the safety and efficiency of CDA with ACDF only focusing on long‐term follow‐ups.

In our meta‐analysis, 11 RCT with more than 5 years’ follow‐up were identified. Based on the quality assessment criteria recommended by the Cochrane Back Review Group^25^, all the studies were rated as low risk of bias. However, blinding to patients and care providers was not appropriately achieved in any studies. In addition, only 4 studies^29^, ^31^, ^32^, ^35^ achieved blinding to outcome evaluators. This may result in reporting bias. Heterogeneity definitely existed in the included studies. First, various different types of CDA devices were used in the 11 RCT, including Kineflex|C^29^, Bryan^30^, ^34^, Discover^35^ Secure‐C^38^, Prestige^28^, ^31^, Mobi‐C^32^, ^37^, ProDisc‐C^33^, and PCM^36^, differing in design and biomechanical properties. Second, the surgical level was different among studies. A total of 8 studies compared one‐level CDD^28^, ^29^, ^30^, ^32^, ^33^, ^34^, ^36^, ^38^, 1 study compared two‐level CDD^31^, and 2 studies compared both one‐level and two‐level CDD^35^, ^37^. Third, the region of studies was also different. Eight studies^28^, ^29^, ^31^, ^33^, ^34^, ^36^, ^37^, ^38^ were conducted in the US and just 3 studies^30^, ^32^, ^35^ were out of the USA. Fourth, evaluation criteria of outcome measures varied among studies. Thus, we performed a sensitivity analysis including comparing two different effect models, using the leave‐one‐out method^27^ and subgroup analysis to find the origin of heterogeneity. The combined results of radiological superior and inferior ASD were not stable and reliable and should be considered with caution. One possible reason is that only 2 studies reported this outcome^36^, ^37^. Although no publication bias existed in neurological success, publication bias existed in secondary surgery at the adjacent level.

After 5 years’ follow up or more, our study revealed that CDA achieved a higher rate of clinical success and better functional outcome measurements with statistical significance, except for NDI score. A mid‐term to long‐term meta‐analysis conducted by Hu *et al*.^17^ compared 4–7 years’ clinical results, pooling data from 8 RCT, and showed that CDA achieved a significantly higher clinical success rate and better functional outcome. Similarly, Gao *et al*.^5^ compared 2–5 years’ clinical results, pooling data for 14 RCT for analysis, and found that CDA was superior in VAS pain scores and neurological success, but NDI scores remained similar. In addition, major functional outcome measurements of CDA proved to have no obvious benefits when pooling 1–2 years’ data into the analysis^24^. This difference may originate from the different follow‐up duration. Theoretically, CDA shares the same procedure of discectomy, endplate preparing, and decompression. VAS arm pain should be similar. However, VAS arm pain score was favored for CDA at the final follow up.

Adverse events are another major concern when applying CDA. Our results showed no statistical difference in total reported AE, serious AE, and device or surgery‐related serious AE. This finding is consistent with some previous meta‐analyses^5^, ^18^, ^23^ but contrary to others^17^. This difference can be explained by the different inclusion criteria for each study. Our study was focused on the long‐term data and only enrolled RCT with more than 5 years’ follow‐up. Undeniably, pseudoarthrosis would not occur after CDA, but heterotopic ossification and bone loss became new problems^12^, ^14^. A recent systematic review^14^ showed that the long‐term heterotopic ossification rate after CDA was 53.6% and the severe (grade 3 and 4) heterotopic ossification rate was 47.5%. In addition, the severe heterotopic ossification rate was significantly associated with follow‐up time, with a 0.63% increase per month growth^14^. Bone loss was as high as 60.4%, although it did not affect mid‐term to long‐term clinical outcomes^12^. This might be the reason why surgeons did not feel confident recommending CDA as a standard option^30^. Moreover, it could explain the similar incidence of AE between CDA and ACDF.

Adjacent segment degeneration is the most important factor to be considered. The initial purpose of designing CDA was to prevent ASD after surgery. The biomechanical advantages have been well established^3^, ^49^. A recent meta‐analysis showed that there was no statistically significant difference in ASD between CDA and ACDF within 24‐months’ follow‐up period, but ASD was significantly lower with an increase of follow‐up duration in CDA^16^. In contrast, Xu *et al*.^21^ and Zhu *et al*.^23^ found that CDA was superior in reducing the ASD incident rate when compared with ACDF, and this superiority became more apparent over time^21^. Although these 3 studies^16^, ^21^, ^23^ attempted to evaluate ASD and symptomatic ASD separately, the follow‐up period was not separated clearly, and long‐term results were weak. Our results show that CDA has significantly lower symptomatic ASD. However, when we pooled all data together, there was no statistical difference in radiological superior ASD between CDA and ACDF. Interestingly, Ren *et al*.^20^ found that ASD was not significantly different between CDA and ACDF with a smaller sample. Nunley *et al*.^50^ (2018) summarized biomechanical and clinical evidence from worldwide application of CDA and concluded that CDA decreased the rate of radiographic adjacent segment pathology by alleviating adjacent‐level stress. However, the reason why subgroup analysis showed no significant difference in the non‐US group is still difficult to explain.

Increased attention has been focused on the secondary surgery rate. Ghobrial *et al*.^40^ found that fewer patients with the Bryan disc required surgery for symptomatic ASD when compared with ACDF without statistical significance at 10 years’ follow‐up. However, they performed combined analysis using Bryan and Prestige artificial discs and found significant differences in symptomatic ASD requiring surgery as early as after 7 years^40^. Surprisingly, MacDowall *et al*.^51^ conducted a retrospective study based on a Swedish database and found that CDA had a similar secondary surgery rate at the adjacent level but a higher secondary surgery rate at the index level with significant difference. However, based on our long‐term results, CDA had a significantly lower rate of total secondary surgery, secondary surgery at the adjacent level, and secondary surgery at the index level, which is consistent with mid‐term to long‐term results^17^ However, this finding is contrary to the short‐term to mid‐term result reported by Zhang *et al*.^52^ that the secondary surgery rate at the adjacent level showed no significant difference. It seems that CDA exhibited superiority in reducing secondary surgery through restoring favorable physiological biomechanical properties in the long‐term follow‐up. However, it is important to note that our subgroup analysis also showed no statistical difference in the secondary surgery rate in the non‐US group.

Several limitations may exist in this study. First, due to our focus on long‐term results, only 11 RCT were included and 8 of them were conducted in the USA. Therefore, our study may not reflect the worldwide results and may result in bias. In addition, larger size samples are needed in future studies. Second, although all included studies were rated as low risk of bias based on the Cochrane Back Review Group, all of them failed to achieve sufficient blinding and the allocation concealment was rarely clearly described. Third, high heterogeneity exists in NDI score, radiological superior ASD and inferior ASD. Our sensitivity analysis results revealed that radiological superior ASD and inferior ASD were not stable and, therefore, should be considered with caution. Finally, subgroup analysis showed different results for NDI score, symptomatic ASD, total secondary surgery, secondary surgery at the index level, and secondary surgery at the adjacent level between US and non‐US regions. Therefore, well‐designed worldwide multi‐center RCTs with long‐term follow‐ups are still needed for further evaluation in the future.

### *Conclusion*

Our study provided further evidence that CDA is superior in achieving long‐term clinical outcomes such as overall success, NDI success and neurological success, VAS neck pain and arm pain, SF‐36 PCS and MCS, symptomatic ASD, total secondary surgery, and secondary surgery at the index level and at the adjacent level. However, no clear benefit could be identified in regard to NDI score, total reported AE, serious AE, device/surgery‐related AE, and radiological superior and inferior ASD. Well‐designed worldwide RCT with long‐term follow up are still necessary for further evaluation in the future.
